# Gemtuzumab ozogamicin in first-line treatment of CBF-AML: insights from a retrospective multi-center analysis

**DOI:** 10.1038/s41375-025-02700-9

**Published:** 2025-07-21

**Authors:** Julian Ronnacker, Philippe J. Muller, Jan-Henrik Mikesch, Sven Zukunft, Barbora Weinbergerová, Jiří Šrámek, Jan Valka, Jan Novak, Pavel Zak, Tomas Szotkowski, Zdenek Koristek, Carolin Krekeler, Julia M. Unglaub, Tim Sauer, Leo Ruhnke, Sabrina Kraus, Judith Schaffrath, Lutz P. Müller, Sabrina Kaes, Dirk Niemann, Lars Fransecky, Patrick P. Hess, Martina Crysandt, Edgar Jost, Joana Millo, Johannes Gaertner, Roland Repp, Madlen Jentzsch, Lea Hoppe, Stefan Klein, Franziska Modemann, Nina Michalowski, Klaudia Fischbach, Wolfgang Blau, Marion Ruhs, Markus Ritter, Julian Lohmeyer, Björn Steffen, Sarah Hauser, Martin Kaufmann, Stefan W. Krause, Ricarda Knabe, Karsten Spiekermann, Hubert Serve, Uwe Platzbecker, Claudia D. Baldus, Carsten Müller-Tidow, Georg Lenz, Hans Christian Reinhardt, Jirí Mayer, Martin Bornhäuser, Christoph Röllig, Christoph Schliemann, Maher Hanoun

**Affiliations:** 1 Department of Medicine A, University Hospital Münster, Münster, Germany; 2 West German Cancer Center (WTZ), Essen-Münster, Germany; 3 Department of Hematology and Stem Cell Transplantation, University Hospital Essen, Essen, Germany; 4 Department of Emergency Medicine, Charité – Universitätsmedizin Berlin, Campus Benjamin Franklin, Berlin, Germany; 5 Department of Internal Medicine I, University Hospital TU Dresden, Dresden, Germany; 6 Department of Internal Medicine - Hematology and Oncology, University Hospital Brno and Masaryk University, Brno, Czech Republic; 7 Department of Hematology and Oncology, University Hospital Pilsen, Pilsen, Czech Republic; 8 Institute of Hematology and Blood Transfusion, Prague, Czech Republic; 9 University Hospital Kralovske Vinohrady and Third Faculty of Medicine, Charles University, Prague, Czech Republic; 10 Department of Hematology, University Hospital Hradec Kralove, Hradec Kralove, Czech Republic; 11 Department of Hematology, Palacky University Olomouc and University Hospital Olomouc, Olomouc, Czech Republic; 12 Department of Hematooncology, Faculty of Medicine, University Hospital Ostrava, Ostrava, Czech Republic; 13 Department of Hematology, Oncology and Rheumatology, University Hospital Heidelberg, Heidelberg, Germany; 14 Department of Internal Medicine II, University Hospital Würzburg, Würzburg, Germany; 15 Department of Hematology and Oncology, University Hospital Halle, Halle, Germany; 16 Department of Internal Medicine, Hematology and Oncology, Gemeinschaftsklinikum Mittelrhein, Koblenz, Germany; 17 Department of Internal Medicine II, University Hospital Schleswig-Holstein, Kiel, Germany; 18 Department of Internal Medicine IV, University Hospital RWTH Aachen, Aachen, Germany; 19 Department of Hematology, Oncology and Palliative Medicine, Rems-Murr-Kliniken, Winnenden, Germany; 20 Department of Internal Medicine 5, Klinikum Nürnberg, Nürnberg, Germany; 21 Department of Internal Medicine 2, Städtisches Krankenhaus Kiel, Kiel, Germany; 22 Department for Internal Medicine I, University Hospital Leipzig, Leipzig, Germany; 23 Department of Hematology and Oncology, University Hospital Mannheim, Mannheim, Germany; 24 Department of Oncology and Hematology, University Hospital Hamburg-Eppendorf, Hamburg, Germany; 25 Hospital Pharmacy, University Hospital Hamburg-Eppendorf, Hamburg, Germany; 26 Department for Hematology, Oncology and Palliative Care, Helios Dr Horst Schmidt Kliniken, Wiesbaden, Germany; 27 Department of Hematology and Oncology, Kliniken Sindelfingen, Sindelfingen, Germany; 28 Department of Hematology and Oncology, University Hospital Frankfurt am Main, Frankfurt am Main, Germany; 29 Department of Hematology, Oncology and Palliative Medicine, Robert-Bosch-Hospital, Stuttgart, Germany; 30 Department of Medicine 5 – Hematology and Oncology, University Hospital Erlangen, Erlangen, Germany; 31 Department of Medicine III, University Hospital LMU Munich, Munich, Germany

**Keywords:** Acute myeloid leukaemia, Acute myeloid leukaemia

## Abstract

The addition of gemtuzumab ozogamicin (GO) to intensive chemotherapy (IC) has become a mainstay in treating patients with core binding factor acute myeloid leukemia (CBF-AML). However, evidence for the efficacy of GO in this particular subgroup is primarily based on meta-analytic data from different trials conducted more than a decade ago. In this registry-based study, we evaluated the impact of adding GO to IC in 265 CBF-AML patients from the SAL, AMLCG, and CELL cooperative study groups. Patients receiving GO had a 2-year overall survival of 90% compared with 80% in those without GO (hazard ratio [HR] 0.45, 95% confidence interval [CI] 0.21–0.95, *P* = 0.036) and a 2-year event-free survival of 51% versus 36% (HR 0.69, 95% CI 0.48–0.99, *P* = 0.046). While complete remission rates in GO vs. non-GO patients were comparable (89% vs. 90%, *P* = 0.81), more GO patients achieved measurable residual disease-negative remission (77% vs. 49%, *P* < 0.001), resulting in numerically reduced cumulative incidence of relapse (HR 0.67, 95% CI 0.43–1.02, *P* = 0.06). Despite delayed platelet recovery, high-grade toxicities were not increased in GO-treated patients. These findings support the integration of GO into treatment protocols for IC-eligible patients with CBF-AML.

## Introduction

Gemtuzumab ozogamicin (GO) is an antibody-drug conjugate that targets CD33, a marker expressed on most acute myeloid leukemia (AML) cells. It combines a CD33 monoclonal antibody with the cytotoxic agent calicheamicin and was first approved in 2000. However, concerns about increased induction mortality led to its market withdrawal in 2010 [1, 2]. In the AML15 trial, which enrolled 137 core-binding factor AML (CBF-AML) patients, 72 of whom received GO, Burnett et al. were the first to demonstrate an improvement in overall survival (OS), particularly in favorable-risk AML, when GO was added to induction [3]. Following the introduction of dose fractionation, the ALFA-0701 trial showed significantly prolonged event-free survival (EFS) and relapse-free survival (RFS), as well as a numerically longer OS, in 271 treatment-naïve AML patients receiving GO in addition to intensive chemotherapy (IC) [4]. A meta-analysis by Hills et al. pooled data from five randomized trials with a total of 3325 AML patients, revealing an OS benefit for the addition of GO [5]. Consistent with the AML15 trial results, the meta-analysis highlighted a pronounced efficacy in patients with cytogenetically favorable AML. It reported a 22.5% absolute OS benefit at 5 years in the subgroup with CBF-AML compared to IC alone. As a result, GO was re-approved by the FDA in 2017 and the EMA in 2018 for the first-line treatment of CD33-expressing AML [2], leading to its incorporation into standard induction regimens, particularly for patients with CBF-AML harboring the t(8;21)(q22;q22.1)/*RUNX1::RUNX1T1* or inv(16)(p13.1q22)/t(16;16)(p13.1;q22)/*CBFB::MYH11* fusion genes [6–8].

Although many centers worldwide have adopted the practice of adding GO to induction chemotherapy in patients with CBF-AML, the relatively modest OS in non-GO patients with favorable cytogenetics in the meta-analysis (55% at five years), along with the variability in IC regimens and inconsistent use of high-dose cytarabine consolidation, remain subjects of debate. Here, to further assess the potential benefit of GO in both young and elderly adult CBF-AML, we retrospectively analyzed data from 265 CBF-AML patients who received first-line IC with or without GO, registered in the databases of the German Study Alliance Leukemia (SAL), the German AML Cooperative Group (AMLCG), and the Czech Leukemia Study Group for Life (CELL).

## Materials and methods

A database search of the registries of the German Study Alliance Leukemia (SAL), the AML Cooperative Study Group (AMLCG), the Czech Leukemia Study Group (CELL), and the University Hospital of Hamburg-Eppendorf identified all adult patients ≥18 years with first diagnosis of CBF-AML registered between January 2014 and December 2023. Patients provided written consent at their respective AML centers, and the registry studies were approved by the ethics committees of the participating study groups.

### Endpoints

Primary endpoints were OS, EFS, and the cumulative incidence of relapse (CIR) in patients achieving composite complete remission (CR). Composite CR included CR and CRi (CR with incomplete hematological recovery). CRi was defined as CR with residual neutropenia <1.0/nl or thrombocytopenia <100/nl [6]. Induction failure was defined as failure to achieve composite CR after 1–2 cycles of induction therapy.

Following current European LeukemiaNet (ELN) guidelines, OS was defined as the time from diagnosis to death [6]. EFS was calculated from the date of diagnosis to the date of induction failure, morphological relapse, molecular failure, or death. CIR was calculated from the date of composite CR to either morphological relapse or molecular failure, and death without relapse or molecular failure was considered a competing risk. Molecular failure was defined as failure to achieve a <3 log_10_ reduction of the transcript by qPCR, conversion from undetectable to detectable, or a ≥1 log_10_ increase according to ELN definitions [9]. Secondary endpoints included achievement of measurable residual disease (MRD)-negative remission, time to MRD negativity, safety, and toxicity. Time to MRD negativity was defined as the time from the start of induction therapy to MRD negativity by PCR. MRD testing was performed in accredited, non-centralized laboratories using standardized quantitative PCR assays (sensitivity 10^−^⁴–10^−^⁵), in accordance with established protocols [10–12].

Safety and toxicity analyses included the time to platelet (≥50/nl) and ANC (≥0.5/nl) recovery after first induction, as well as the occurrence of sinusoidal obstruction syndrome (SOS) of any grade, hemorrhage Common Terminology Criteria of Adverse Events (CTCAE) grade ≥3, and infections CTCAE grade ≥3. Bleeding and infections were analyzed during the first induction only.

### Statistical analysis

Time-to-event outcomes were estimated using the Kaplan–Meier method, and differences between groups were assessed with the log-rank test. Cumulative incidence functions for competing risks were compared using Gray’s test. Patient characteristics were analyzed with the Mann–Whitney U test for continuous variables and either Fisher’s exact test or the chi-square test for categorical variables, as appropriate. Multivariable analysis was performed using a Cox proportional hazards regression model. For competing risks endpoints, a regression model according to Fine and Gray was applied [13]. All tests were two-tailed, and a *P*-value < 0.05 was considered statistically significant. All analyses were performed using RStudio (version 2023.12.1).

## Results

### Patient characteristics

A total of 265 CBF-AML patients diagnosed in the participating sites were identified and included in the analysis. Amongst those, 80 patients received GO at a median (range) cumulative dose of 9 (3-9) mg/m^2^ as part of induction chemotherapy, and 185 patients receiving induction therapy without GO served as the non-GO control group. Patient characteristics of the GO and non-GO cohorts are shown in Table 1. The majority of patients were diagnosed with de novo CBF-AML (90% vs. 91%). There were no significant differences between the two groups with respect to median age (GO vs. non-GO patients, 47 vs. 50 years), female sex (43% vs. 50%), or ECOG performance status (ECOG ≤1, 92% vs. 89%) at the time of diagnosis. In the GO and non-GO groups, 41% and 45% of patients, respectively, harbored the *RUNX::RUNX1T1* fusion gene. Both cohorts were also similar in terms of other AML characteristics at the time of diagnosis, including white blood cell (WBC) count, peripheral blood and bone marrow blasts, lactate dehydrogenase (LDH) levels, additional cytogenetic aberrations otherwise considered adverse according to ELN criteria, and selected co-mutations, including *KIT*, *FLT3*-ITD and *NRAS* mutations. The frequency of *FLT3*-TKD as well as of other co-mutations was higher in GO versus non-GO patients (*FLT3*-TKD, 18% vs. 7%, *P* < 0.001; other co-mutations, 34% vs. 16%, *P* < 0.001), which however, may relate to the fact that GO patients were treated at a later time point when a more comprehensive molecular panel became standard of care based on updated guidelines (2018 or later).

**Table 1:** Baseline and treatment-related characteristics of GO- and non-GO-treated patients.

	GO (*n* = 80)	Non-GO (*n* = 185)	*P*
Baseline characteristics			
Age at first diagnosis, years, median (range)	47 (20–75)	50 (19–82)	0.51^a^
Female sex, n (%)	34 (43)	93 (50)	0.25^b^
ECOG, n (% of available)			
0 or 1	69 (92)	141 (88)	0.37^b^
≥2	6 (8)	19 (12)	
AML characteristics			
WBC, /nl, median (range)	12.1 (1.4–273)	17.2 (1.4–243.4)	0.43^b^
Peripheral blasts, %, median (range)	50 (0–95)	36 (0–98)	0.08^a^
Bone marrow blasts, %, median (range)	60 (5-95)	52 (3–91)	0.65^a^
LDH, U/l, median (range)	454 (216–5411)	558.5 (64–3497)	0.14^a^
AML ontogeny, n (%)			
De novo	72 (90)	169 (91)	0.75^c^
tAML	8 (10)	15 (8)	
sAML	0 (0)	1 (1)	
CBF fusion gene, n (%)			
*RUNX1::RUNX1T1*	33 (41)	83 (45)	0.59^b^
*CBFB::MYH11*	47 (59)	102 (55)	
Presence of other cytogenetic aberrations, n (% of available)	33 (42)	75 (42)	0.09^b^
Additional adverse cytogenetics according to ELN 2022, n (% of available)	4 (5)	7 (4)	0.74^c^
Co-mutations, n (%)			
*c-KIT*	15 (19)	31 (17)	0.69^b^
*FLT3*-TKD	14 (18)	13 (7)	**<0.001**^**b**^
*FLT3*-ITD	3 (4)	8 (4)	1.00^c^
*NRAS*	10 (13)	13 (7)	0.15^b^
Others	27 (34)	29 (16)	**<0.001**^**b**^
Treatment characteristics			
Number of induction cycles, median (range)	1 (1–2)	1 (1–2)	**0.025**^**a**^
Cumulative GO dosage during induction, median (range), mg/m^2^ (maximum of 5 mg absolute)	9 (3–9)	-	
Induction regimen, n (%)			
7 + 3	80 (100)	159 (86)	**<0.001**^**c**^
Others^d^	0 (0)	26 (14)	
Number of consolidation cycles, median (range)	3 (2–4)	3 (1–4)	0.47^a^
Number of patients with GO in consolidation, n (%)	25 (36)	-	
Consolidation backbone, n (%)			
IDAC	28 (37)	42 (26)	0.07^c^
HiDAC	46 (61)	106 (67)	
Others^e^	1 (1)	11 (7)	
Composite CR rate (CR/CRi) after induction therapy, n (%)	71 (89)	166 (90)	0.81^b^
Allogeneic HCT, n (%)	28 (35)	90 (49)	**0.040**^**b**^
CR1	18 (64)	56 (62)	0.93^b^
≥ CR2	5 (18)	15 (17)	
Active disease	5 (18)	19 (21)	

*AML* acute myeloid leukemia, *CBF* core-binding factor, *CR* complete remission, *CRi* CR with incomplete hematological recovery, *ECOG* Eastern Cooperative Oncology Group performance status, *ELN* European LeukemiaNet, *GO* gemtuzumab ozogamicin, *HCT* hematopoietic cell transplantation, *HiDAC* high-dose cytarabine, *IDAC* intermediate-dose cytarabine, *LDH* lactate dehydrogenase, *sAML* secondary AML, *tAML* therapy-related AML, *WBC* white blood count.

Significant *P* values are in bold.

^a^Mann–Whitney U test.

^b^chi-squared test.

^c^Fisher’s exact test.

^d^Includes cytarabine/anthracycline (*n* = 23), 6-thioguanine/cytarabine/daunorubicin (TAD-9, *n* = 2), CPX-351 (*n* = 1).

^e^Includes azacitidine/venetoclax (*n* = 1) for GO patients and 6-thioguanine/cytarabine/daunorubicin (TAD-9, *n* = 9), low-dose cytarabine/anthracycline (*n* = 2) for non-GO patients.

Patients received a median number of one induction cycle in both groups. In the GO group, all patients received standard 7 + 3 cytarabine/daunorubicin as chemotherapy backbone, and 8 patients (10%) additionally received the kinase inhibitor midostaurin. The median (range) GO dose was 9 (3-9) mg/m^2^, with 19 patients (24%) receiving <3 doses of GO in induction. In the non-GO group, 159 patients (86%) received 7 + 3, with 7 patients (4%) also receiving midostaurin. Twenty-six (14%) non-GO patients received other induction regimens, including 23 patients who received high-dose cytarabine and mitoxantrone (HAM).

A median number of 3 consolidation cycles was administered in both groups. Twenty-five (36%) patients in the GO cohort received GO also in consolidation. In the GO cohort, 28 (37%) and 46 (61%) of patients received intermediate-dose (IDAC) and high-dose cytarabine (HiDAC), respectively, and 15 (20%) patients received anthracycline-containing consolidation, following a consolidation strategy that was used in the ALFA-0701 trial [14]. In the non-GO group, 106 (67%) received HiDAC and 42 (26%) received IDAC. Twenty-one (13%) non-GO patients received an anthracycline during consolidation, including 9 patients receiving TAD and 2 patients receiving lower doses of cytarabine in combination with daunorubicin. A total of 4 (5%) and 8 (5%) patients received midostaurin during consolidation in the GO and non-GO cohort, respectively.

Composite CR rates after induction therapy were similar in the GO (89%) and non-GO (90%) groups (*P* = 0.81). In the GO group, 28 (35%) patients underwent allogeneic hematopoietic cell transplantation (HCT) vs. 90 (49%) in the non-GO group (*P* = 0.040). The proportion of patients transplanted either in CR1, ≥CR2, or with active disease was similar in GO and non-GO patients (Table 1).

### Primary endpoints

**Fig. 1:**
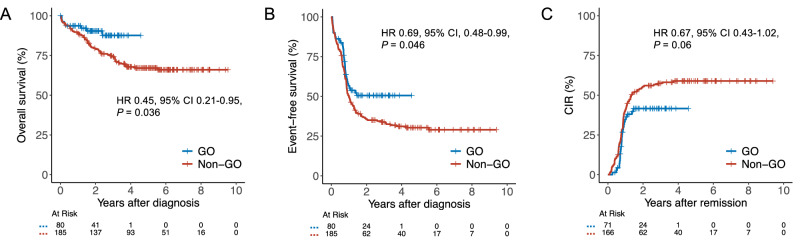
Time-to-event outcomes in GO- vs. non-GO-treated patients. The addition of GO was associated with significantly improved OS (**A**) and EFS (**B**). CIR was numerically lower in GO-treated patients compared to those who did not receive GO (**C**), although the difference did not reach statistical significance. GO gemtuzumab ozogamicin, HR hazard ratio, CI confidence interval.

Median (range) follow-up was 2.2 (0.4–4.6) and 5.5 (0.3–9.5) years in the GO and non-GO cohorts, respectively. OS was significantly longer in the GO cohort compared to the non-GO cohort (HR 0.45; 95% CI 0.21–0.95; *P* = 0.036; Fig. 1A) with 2-year OS estimates of 90% (95% CI, 84–98%) vs. 80% (95% CI, 74–86%). Similarly, estimated EFS was significantly improved in GO vs. non-GO-treated patients (HR 0.69, 95% CI, 0.48–0.99, *P* = 0.046, Fig. 1B), with 2-year EFS estimates of 51% (95% CI, 40–64%) vs. 36% (95% CI, 29–43%). CIR showed a numerical benefit in favor of the GO group (2-year estimate 42% (95% CI, 29–54%) vs. 55% (95% CI, 48–63%) (HR 0.67, 95% CI 0.43–1.02, *P* = 0.06; Fig. 1C)). Two-year non-relapse mortality was 1% (95% CI, 0–4%) vs. 5% (95% CI, 2–8%) in GO vs. non-GO patients (HR 4.05, 95% CI, 0.53–30.9, *P* = 0.18). Thus, EFS differences in GO vs. non-GO patients were primarily driven by an increased incidence of relapse.

**Fig. 2:**
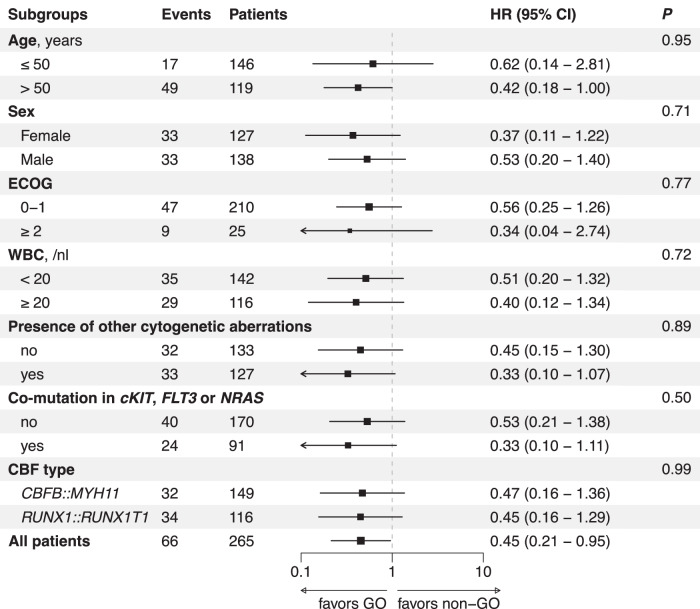
Subgroup analysis of the effect of GO treatment on overall survival. No heterogeneity in treatment effect was detected across subgroups, as assessed by tests for interaction. Missing values for ECOG, WBC, karyotype aberrations, and co-mutations were not imputed. CBF core-binding factor, ECOG Eastern Cooperative Oncology Group performance score, GO gemtuzumab ozogamicin, HR hazard ratio, CI confidence interval, WBC white blood count.

In multivariable analyses adjusting for age, sex, AML ontogeny, CBF fusion gene, and additional cytogenetic or molecular alterations, treatment with GO remained associated with a favorable OS (HR_adjusted_ 0.42, 95% CI 0.19–0.92, *P* = 0.031) and EFS (HR_adjusted_ 0.65, 95% CI 0.44–0.96, *P* = 0.031*;* Table 2), while CIR was non-significantly reduced in GO vs. non-GO patients (HR_adjusted_ 0.66, 95% CI 0.42–1.04, *P* = 0.07). There was no significant heterogeneity of GO treatment effects on OS among patients with different baseline characteristics, including age (≤50 and >50 years), sex, ECOG performance status, WBC at diagnosis (<20 and ≥20/nl), CBF fusion gene, or the presence of additional cytogenetic or molecular alterations (Fig. 2). Survival curves stratified by CBF fusion genes are shown in Supplementary Fig. 1. Notably, survival outcomes were similar regardless of whether GO patients received 1 or 2 versus 3 doses of GO during induction (HR 0.48, 95% CI, 0.06–3.90, *P* = 0.49), suggesting consistent efficacy across dosing regimens. However, this finding should be interpreted with caution, given the small sample size and the potential bias introduced by dose reductions due to early toxicity.

**Table 2:** Multivariable analysis for primary endpoints.

	OS			EFS				CIR		
	HR	95% CI	*P*	HR	95% CI	*P*		HR	95% CI	*P*
Therapy										
Non-GO	Ref.			Ref.				Ref.		
GO	0.42	0.19–0.92	**0.031**	0.65	0.44–0.96	**0.031**		0.66	0.42–1.04	0.07
Sex										
Male	Ref.			Ref.				Ref.		
Female	1.00	0.61–1.67	0.98	1.01	0.73–1.41	0.93		0.91	0.61–1.33	0.61
Age, per 10-year increase	1.77	1.44–2.17	**<0.001**	1.16	1.04–1.31	**0.010**		1.08	0.94–1.23	0.30
AML ontogeny										
De novo	Ref.			Ref.				Ref.		
Secondary-type	1.01	0.44–2.29	0.99	0.97	0.54–1.76	0.92		0.92	0.43–1.95	0.83
CBF fusion gene										
*CBFB::MYH11*	Ref.			Ref.				Ref.		
*RUNX1::RUNX1T1*	1.47	0.89–2.44	0.13	1.07	0.78–1.48	0.66		0.90	0.62–1.31	0.60
Cytogenetics										
CBF only	Ref.			Ref.				Ref.		
Additional cytogenetic aberrations	0.77	0.46-1.28	0.31	1.09	0.79-1.51	0.59		1.17	0.80-1.72	0.42
Mutations										
No additional mutations	Ref.			Ref.				Ref.		
Additional mutation in *c-KIT*, *NRAS,* or *FLT3*	1.44	0.86–2.41	0.17	1.26	0.90–1.76	0.17		1.10	0.74–1.63	0.65

*AML* acute myeloid leukemia, *CBF* core-binding factor, *CI* confidence interval, *CIR* cumulative incidence of relapse, *EFS* event-free survival, *GO* gemtuzumab ozogamicin, *HR* hazard ratio, *OS* overall survival.

Significant *P* values are in bold.

To address potential selection bias, specifically the possibility that GO was predominantly administered to fitter patients, we compared the outcomes of non-GO patients diagnosed before (*n* = 114) and after (*n* = 71) the re-approval of GO in April 2018. Although a higher proportion of non-GO CBF-AML patients after April 2018 received IDAC vs. HiDAC, there were no significant differences between these two groups with respect to established or potential risk factors such as age, comorbidities, presence of other cytogenetic aberrations or co-mutations (Supplementary Table 1), nor with respect to OS (HR 0.95, 95% CI 0.56–1.61, *P* = 0.84; Supplementary Fig. 2). Of note, the number of CBF-AML patients not treated with GO diminished after the end of 2019. In a further sensitivity analysis, excluding the 9 (GO) and 10 (non-GO) patients receiving concomitant therapy with a KIT inhibitor either during induction or consolidation, GO patients continued to have a favorable OS compared to non-GO patients (HR 0.50, 95% CI, 0.20–1.01, *P* = 0.05).

### Time to MRD negativity

Seventy (88%) patients in the GO cohort and 158 (85%) patients in the non-GO cohort achieved a composite CR and had evaluable MRD data. A total of 77% (54/70) of evaluable GO patients achieved MRD-negative remission at any time point after induction therapy compared with 49% (77/158) of evaluable non-GO patients (*P* < 0.001). The median time to MRD negativity was significantly shorter in GO vs. non-GO patients at 5.6 (1-23) vs. 8.4 (0-85) months (*P* < 0.001). No difference in overall MRD negativity rates was observed in patients who received GO only during induction or also during consolidation therapy (74 vs. 75%, *P* = 0.92), while the time to MRD-negative remission was 5.7 vs. 4.4 months (*P* = 0.23).

### Safety and toxicity

Analyses included the time to platelet and ANC recovery after first induction, as well as the occurrence of selected adverse events. Data on platelet and ANC recovery were available for 199 (75%) and 198 (75%) patients with CR/CRi after induction therapy, respectively. GO treatment was associated with a significantly longer time to platelet recovery of 2 days (GO vs. non-GO, median days 24 vs. 22, *P* < 0.001), whereas the median time to ANC recovery was identical in both groups (26 vs. 26 days, *P* = 0.48; Supplementary Table 2). SOS was observed in 4 (5%) patients treated with GO compared to 2 (1%) in the non-GO group. All patients with reported SOS had received more than one dose of GO. No cases of SOS-related deaths were reported. Higher-grade bleeding events CTCAE grade ≥3 occurred in 9 (11%) and 15 (8%), respectively, and infections grade ≥3 after first induction occurred in 41 (51%) and 87 (49%) of the GO and non-GO patients, respectively (Supplementary Table 3).

## Discussion

For decades, the combination of cytarabine and daunorubicin has been the cornerstone of AML induction therapy. The use of the 7 + 3 protocol followed by high-dose cytarabine consolidation in CBF-AML has demonstrated remission rates of approximately 90% and 3- and 5-year OS rates of 65–70% and 58–65%, respectively [15–19]. In the meta-analysis by Hills et al., the addition of GO to IC provided the greatest benefit in AML patients with favorable (i.e., CBF) cytogenetics, with a 5-year OS probability of 78% versus 55% [5]. While the difference in OS between GO and non-GO-treated patients was encouraging, some limitations of the meta-analysis should be considered. Notably, only 7% of the included patients had a CBF fusion gene, and the overall results were largely driven by the AML15 trial [3]. Moreover, survival in non-GO patients was comparatively low relative to other contemporary studies of IC-eligible CBF-AML patients [16–18, 20], possibly reflecting heterogeneity or suboptimal chemotherapy backbones in some of the included trials. Despite these limitations, however, the addition of GO to induction therapy has gained broad acceptance in the treatment of CBF-AML.

Against this background, our multicenter retrospective analysis of 265 CBF-AML patients confirmed a significantly longer OS of 90% vs. 80% at 2 years with the addition of GO to IC induction, adding an OS benefit of approximately 10%. Our data align with previous reports showing improved outcomes following the addition of GO in CBF-AML patients [1, 3, 5, 21]. Notably, survival outcomes of non-GO patients in our cohort are consistent with previously reported data on CBF-AML [16–18], supporting the validity of our analysis. While remission rates were comparable between GO-treated and non-treated patients, GO treatment resulted in a higher proportion of MRD-negative remissions and a numerically reduced incidence of AML relapse. These findings are consistent with two recent studies that have highlighted the benefits of GO in terms of enhanced MRD clearance and reduced relapse risk [22, 23]. In contrast, a recent multicenter retrospective analysis in a cohort of 200 CBF-AML patients reported comparable OS for treatment regimens with and without GO [24]. Differences in treatment protocols, particularly chemotherapy backbones and GO dosage, may account for these divergent findings.

Both SOS and prolonged myelotoxicity have been associated with GO [1, 4, 14, 25]. Indeed, our study confirmed a significant delay in platelet recovery by two days, which did not translate into an increased incidence of clinically significant bleeding. Consistent with previous studies, we found a numerically increased risk of SOS among patients treated with GO, which did not adversely affect survival outcomes.

Our results provide compelling evidence supporting the feasibility of incorporating GO into induction therapy for CBF-AML, although some limitations should be acknowledged. First, the retrospective nature of our study introduces a potential risk of selection bias. While patient characteristics, particularly age and performance status, did not differ significantly among patients who were or were not treated with GO after its re-approval in April 2018, and OS among non-GO patients did not differ significantly when comparing those treated before versus after the reintroduction of GO, we cannot fully exclude the possibility of unmeasured confounding. Temporal changes in clinical practice, such as the sporadic use of small-molecule inhibitors or advances in supportive care, may have influenced outcomes. Notably, our sensitivity analysis, which excluded patients treated with KIT inhibitors at any point during therapy, still demonstrated a benefit associated with GO, suggesting that the observed effect was not significantly confounded by KIT inhibitor use. Second, although MRD assessments were conducted using similar techniques and sensitivity thresholds, the analyses were not centralized, and monitoring schedules may have varied across centers. Third, the heterogeneity of consolidation regimens, particularly among non-GO patients, may have impacted outcomes, despite the fact that survival in the non-GO group was consistent with other contemporary CBF-AML cohorts.

To our knowledge, this study represents the largest analysis to date comparing outcomes in intensively treated CBF-AML patients with and without GO. Although follow-up in the GO cohort was relatively short, the observed 2-year survival benefit appears to be primarily attributable to a reduced risk of relapse rather than higher remission rates, consistent with the findings reported by Hills et al. in the favorable-risk cytogenetic subgroup. In a contemporary cohort, our data show an absolute survival advantage of 10% at 2 years with GO-containing induction therapy and acceptable toxicity, thereby supporting the integration of GO into treatment algorithms for patients with CBF-AML.

## Supplementary information

Supplementary data

## Data Availability

The datasets analyzed in the current study are available from the corresponding author upon reasonable request.
