# Supplementary material for: Gemtuzumab ozogamicin in first-line treatment of CBF-AML: insights from a retrospective multi-center analysis
Source: Leukemia. 2025 Jul 21;39(9):2174–80. doi: 10.1038/s41375-025-02700-9 (PMC12380593; doi:10.1038/s41375-025-02700-9)
Supplement: Supplementary file 1 — Supplementary data [file 41375_2025_2700_MOESM1_ESM.pdf]

Gemtuzumab Ozogamicin in First Line Treatment of CBF-AML: Insights from a Retrospective Multi-Center Analysis

Julian Ronnacker[1]*, Philippe J. Muller[2,3]*, Jan-Henrik Mikesch[1], Sven Zukunft[4], Barbora Weinbergerová[5], Jiří Šrámek[6], Jan Valka[7], Jan Novak[8], Pavel Zak[9], Tomas Szotkowski[10], Zdenek Koristek[11], Carolin Krekeler[1], Julia M. Unglaub[12], Tim Sauer[12], Leo Ruhnke[4], Sabrina Kraus[13], Judith Schaffrath[14], Lutz P. Müller[14], Sabrina Kaes[15], Dirk Niemann[15], Lars Fransecky[16], Patrick P. Hess[17], Martina Crysandt[17], Edgar Jost[17], Joana Millo[18], Johannes Gaertner[19], Roland Repp[20], Madlen Jentzsch[21], Lea Hoppe[22], Stefan Klein[22], Franziska Modemann[23], Nina Michalowski[24], Klaudia Fischbach[25], Wolfgang Blau[25], Marion Ruhs[26], Markus Ritter[26], Julian Lohmeyer[27], Björn Steffen[27], Sarah Hauser[28], Martin Kaufmann[27], Stefan W. Krause[29], Ricarda Knabe[30], Karsten Spiekermann[30], Hubert Serve[27], Uwe Platzbecker[21], Claudia D. Baldus[16], Carsten Müller-Tidow[12], Georg Lenz[1], Hans Christian Reinhardt[2], Jirí Mayer[5], Martin Bornhäuser[4], Christoph Röllig[4], Christoph Schliemann[1]* and Maher Hanoun[2]*

[1]University Hospital Münster, Department of Medicine A, Münster, Germany. West German Cancer Center (WTZ) Essen-Münster, Germany,

[2]University Hospital Essen, Department of Hematology and Stem Cell Transplantation, Essen, Germany. West German Cancer Center (WTZ) Essen-Münster, Germany,

[3]Charité – Universitätsmedizin Berlin, Department of Emergency Medicine, Campus Benjamin Franklin, Berlin, Germany,

[4]University Hospital TU Dresden, Department of Internal Medicine I, Dresden, Germany,

[5]University Hospital Brno and Masaryk University, Department of Internal Medicine - Hematology and Oncology, Brno, Czech Republic,

[6]*University Hospital Pilsen, Department of Hematology and Oncology, Pilsen, Czech Republic,*

[7]*Institute of Hematology and Blood Transfusion, Prague, Czech Republic,*

[8]*Charles University, University Hospital Kralovske Vinohrady and Third Faculty of Medicine, Prague, Czech Republic,*

[9]*University Hospital Hradec Kralove, Department of Hematology, Hradec Kralove, Czech Republic,*

[10]*Palacky University Olomouc and University Hospital Olomouc, Department of Hematology, Olomouc, Czech Republic,*

[11]*University Hospital Ostrava, and Faculty of Medicine, Department of Hematooncology, Ostrava, Czech Republic,*

[12]*University Hospital Heidelberg, Department of Hematology, Oncology and Rheumatology, Heidelberg, Germany,*

[13]*University Hospital Würzburg, Department of Internal Medicine II, Würzburg, Germany,*

[14]*University Hospital Halle, Department of Hematology and Oncology, Halle, Germany,*

[15]*Gemeinschaftsklinikum Mittelrhein, Department of Internal Medicine, Hematology and Oncology, Koblenz, Germany,*

[16]*University Hospital Schleswig-Holstein, Department of Internal Medicine II, Kiel, Germany,*

[17]*University Hospital RWTH Aachen, Department of Internal Medicine IV, Aachen, Germany,*

[18]*Rems-Murr-Kliniken, Department of Hematology, Oncology and Palliative Medicine, Winnenden, Germany,*

[19]*Klinikum Nürnberg, Department of Internal Medicine 5, Nürnberg, Germany,*

[20]*Städtisches Krankenhaus Kiel, Department of Internal Medicine 2, Kiel, Germany,*

[21]*University Hospital Leipzig, Department for Internal Medicine I, Leipzig, Germany,*

[22]*University Hospital Mannheim, Department of Hematology and Oncology, Mannheim, Germany,*

[23]*University Hospital Hamburg-Eppendorf, Department of Oncology and Hematology, Hamburg, Germany,*

[24]*University Hospital Hamburg-Eppendorf, Hospital Pharmacy, Hamburg, Germany,*

[25]*Helios Dr Horst Schmidt Kliniken, Department for Hematology, Oncology and Palliative Care, Wiesbaden, Germany,*

[26]*Kliniken Sindelfingen, Department of Hematology and Oncology, Sindelfingen, Germany,*

[27]*University Hospital Frankfurt am Main, Department of Hematology and Oncology, Frankfurt am Main, Germany,*

[28]*Robert-Bosch-Hospital, Department of Hematology, Oncology and Palliative Medicine, Stuttgart, Germany,*

[29]*University Hospital Erlangen, Department of Medicine 5 – Hematology and Oncology, Erlangen, Germany,*

[30]*University Hospital LMU Munich, Department of Medicine III, Munich, Germany*

*These authors contributed equally to this work.

Supplementary Figures

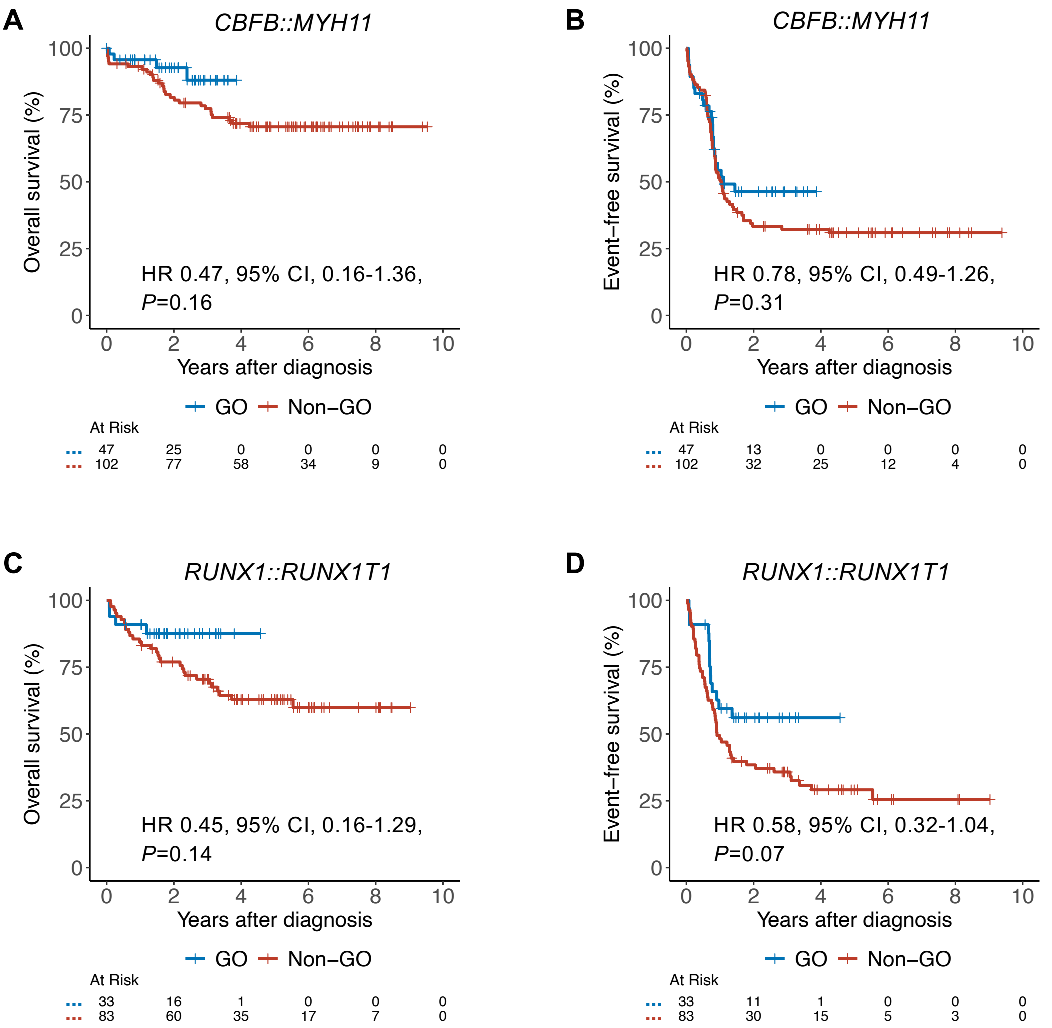

Supplementary Figure 1. Overall survival and event-free survival by cytogenetic subgroup. No heterogeneity in the treatment effect of GO was observed. CI, confidence interval. HR, hazard ratio.

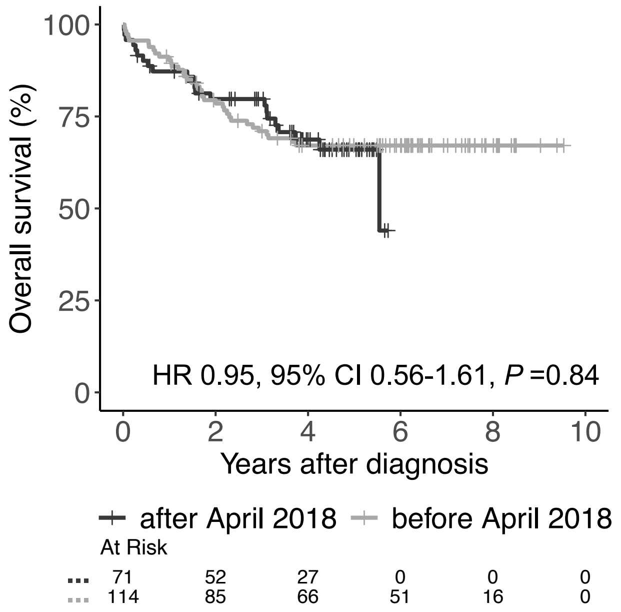

Supplementary Figure 2. Overall survival in non-GO-treated patients before and after the approval of GO in April 2018. No significant difference in survival was observed. CI, confidence interval. HR, hazard ratio

Supplementary Tables

Supplementary Table 1. Characteristics of non-GO patients before and after the approval of GO in April 2018.

	**Non-GO, before 04/2018** (n = 114)	**Non-GO after 04/2018** (n = 71)	***P***
**Baseline characteristics**			
**Age at first diagnosis**, years, median (range)	50 (19-82)	47 (21-75)	0.96[a]
**Female sex**, n (%)	65 (57)	28 (39)	**0.020**[b]
**ECOG**, n (% of n available)			
0 or 1	83 (87)	58 (89)	0.72[b]
≥2	12 (13)	7 (11)	
**AML characteristics**			
**WBC**, /nl, median (range)	16.7 (1.5-228.7)	17.4 (1.4-234.4)	0.41[a]
**Peripheral blasts**, %, median (range)	36 (0-96)	35 (0-96)	0.91[a]
**Bone marrow blasts**, %, median (range)	50 (3-91)	58 (3-90)	0.06[a]
**LDH**, U/l, median (range)	573 (67-3497)	545 (103-3430)	1.00[a]
**AML ontogeny**, n (%)			
De novo	109 (96)	60 (85)	**0.016**[c]
tAML	5 (4)	10 (14)	
sAML	0 (0)	1 (1)	
**CBF fusion gene**, n (%)			
RUNX1::RUNX1T1	49 (43)	34 (48)	0.51[b]
CBFB::MYH11	65 (57)	37 (52)	
**Presence of other cytogenetic aberrations**, n (%)	43 (38)	32 (45)	0.32[b]
Yes	43 (38)	32 (45)	0.44[b]
No	68 (60)	38 (54)	
Missing	3 (3)	1 (1)	
**Additional adverse cytogenetics according to ELN** 2022, n (%)			
Yes	7 (6)	0 (0)	**0.044**[c]
No	104 (91)	70 (99)	
Missing	3 (3)	1 (1)	
**Co-mutations, n (%)**			
Yes	30 (26)	28 (39)	0.10[b]

No	81 (71)	42 (59)	
Missing	3 (3)	1 (1)	
**Treatment characteristics**			
**Number of induction cycles**, median (range)	1 (1-2)	1 (1-2)	0.51[a]
**Induction regimen**, n (%)			
7+3	95 (83)	65 (92)	0.11[b]
Others	19 (17)	6 (8)	
**Number of consolidation cycles**, median (range)	3 (1-4)	3 (1-4)	0.08[a]
**Consolidation backbone**, n (%)			
IDAC	18 (20)	23 (39)	**0.027**[c]
HiDAC	66 (74)	35 (59)	
Others	5 (6)	1 (2)	
**Composite CR rate (CR/CRi) after induction therapy**, n (%)	101 (89)	65 (92)	0.52[b]
**Allogeneic HCT**, n (%)	56 (49)	34 (48)	0.87[b]
CR1	33 (59)	22 (65)	0.18[b]
≥CR2	8 (14)	8 (24)	
Active disease	15 (27)	4 (12)	

[a]Mann Whitney U test
[b]chi-squared test
[x]Fisher's exact test

AML, acute myeloid leukemia. CBF, core binding factor. CR, complete remission. CRi, CR with incomplete hematological recovery. ECOG, Eastern Cooperative Oncology Group performance status. ELN, European LeukemiaNet. GO, gemtuzumab ozogamicin. HCT, hematopoietic stem cell transplantation. HiDAC, high-dose cytarabine. IDAC, intermediate-dose cytarabine. HCT, hematopoietic cell transplantation. LDH, lactate dehydrogenase. sAML, secondary AML. tAML, therapy-related AML. WBC, white blood count.

Supplementary Table 2. Time to platelet and ANC recovery after first induction in GO- vs. non-GO-treated patients.

	GO	Non-GO	*P*
**Time to PLT recovery ≥50/nl**, days, median (range)	24 [11-42]	22 [13-49]	**<0.001**[a]
**Time to ANC recovery ≥0.5/nl**, days, median (range)	26 [15-43]	26 [14-52]	0.48[a]

[a]Mann Whitney U test

ANC, absolute neutrophil count. GO, gemtuzumab ozogamicin. PLT, platelet.

Supplementary Table 3. Selected toxicities in GO- vs. non-GO-treated patients.

	GO	Non-GO	*P*
**SOS,** n (%)	4 (5%)	2 (1%)	0.07[a]
**Hemorrhage ≥CTCAE 3° in first induction**, n (%)	9 (11%)	15 (8%)	0.56[b]
**Infections ≥CTCAE 3° in first induction**, n (%)	41 (51%)	87 (49%)	0.62[b]

[a]Fisher's exact test
[b]chi-squared test

CTCAE, Common Terminology Criteria for Adverse Events. GO, gemtuzumab ozogamicin.

SOS, sinusoidal obstruction syndrome.
